# PLANT vs. PATHOGEN: Enlisting Tobacco in the Fight Against Anthrax

**DOI:** 10.1289/ehp.114-a364

**Published:** 2006-06

**Authors:** Graeme Stemp-Morlock

Just weeks after the September 11 terrorist attacks in New York and Washington, letters containing anthrax spores were mailed to newspapers and television stations in New York and to two U.S. senators on Capitol Hill. Although only a few letters were sent, 22 people were infected and 5 died. More importantly, the bioterror attacks fueled fears that future attacks might be more extensive. Now researchers at the University of Central Florida are helping to prepare for the possibility of anthrax attacks by developing a new technique that can quickly produce hundreds of millions of doses of a potentially safer anthrax vaccine.

Since the 1960s American microbiologists have produced a vaccine for anthrax from the very microbe itself, *Bacillus anthracis*. The microbe’s toxin is made up of three key parts: edema factor (EF), lethal factor (LF), and protective antigen (PA). EF causes fluid to build up in the area of infection, while LF kills cells or prevents them from working. However, both of these factors require PA to create a passageway into the cells—the PA bonds to protein receptors, creating a new complex to which the other two factors attach.

According to Stephen Leppla, a senior researcher at the National Institute of Allergy and Infectious Diseases (NIAID), anthrax bacteria that don’t have PA cannot cause an infection. “In essence,” he says, “they are inactivated and become much less virulent.” The current anthrax vaccine works on this very antibodies are created. PA introduced in the event of a future anthrax exposure would be inactivated by these antibodies, stopping the infection in its tracks.

## In Pursuit of PA

But obtaining large quantities of PA has been a problem. Only one company—BioPort of Lansing, Michigan—is licensed by the FDA to produce the vaccine in the United States, and it can produce only 8 million doses each year through a fermentation process, according to BioPort spokeswoman Kimberly Brenne Root. That’s enough to fill the company’s contracts with the Department of Defense (DOD) and the Department of Health and Human Services, which stockpile the vaccine and administer it to military personnel, but not enough to vaccinate a large civilian population in the event of a widespread attack.

In 2004, in an attempt to procure more doses of vaccine, the U.S. government awarded an $877.5-million contract to VaxGen of Brisbane, California, to produce 75 million doses by the end of 2006. Setbacks have resulted in major delays, however; on 10 May 2006, company officials confirmed that the first shipments of the vaccine won’t be delivered before late 2007 at the very soonest.

As well, there have been concerns that the vaccine produced by BioPort was not safe. Several Gulf War service members reported health problems after being vaccinated. Anecdotal reports suggest the vaccine may contribute to heart problems, cardiovascular illness, seizures, Gulf War syndrome, even death. Documented side effects include pain and swelling at the injection site, inflammation, flu-like symptoms, malaise, rash, joint pain, and headache. The BioPort vaccine can be contaminated with small amounts of LF and EF, which may contribute to the adverse effects associated with it.

To overcome these problems, Henry Daniell, a professor of molecular biology and microbiology at the University of Central Florida, has been on the hunt for a way to produce large quantities of “clean” PA, free of EF and LF. Now he thinks he has finally found it.

## Turning Over a New Leaf

Daniell and his team began by isolating the gene for PA from *B. anthracis*. Then they inserted the gene into tobacco plants. “There are a lot of advantages to tobacco plants,” says Daniell. “They produce a lot of biomass. . . . Also, we didn’t want to produce a vaccine in a food crop in case there was cross-contamination or some package got mixed up on some truck somewhere.” (Although tobacco shipments also could get mixed up, burning the tobacco in the course of smoking would destroy the PA it contained.) Furthermore, Daniell says, “[Tobaccco plants] are very easy to genetically manipulate.”

Daniell’s team chose to insert the gene into the chloroplast rather than the cell nucleus since they could get far more copies of the PA protein that way. After harvesting the tobacco plants, Daniell’s team found that each plant produced about 150 milligrams of anthrax PA. That adds up to 360 million doses’ worth of PA from one acre of tobacco plants. And because only PA is produced, the resulting vaccine will be cleaner than one produced through fermentation.

When the PA was introduced into mice, the rodents responded by producing very high levels of anti-PA antibodies. The immunized mice were sent to the NIAID, where they underwent anthrax toxin challenge. There, Leppla injected the mice with 150 micrograms of anthrax toxin, 1.5 times the amount needed to kill a mouse. Yet, the mice survived, proving that the new technique could produce an effective vaccine. These findings were published in the December 2005 issue of *Infection and Immunity*.

## Practical Considerations

Rakesh Bhatnagar, chairman of the Centre for Biotechnology at Jawaharlal Nehru University in New Delhi, India, has researched plant-based anthrax vaccines, and has signed a commercial agreement to produce larger quantities of anthrax PA than BioPort while still using a fermentation system. He believes Daniell’s research is significant because it shows that PA produced in plants can protect animals from anthrax. Yet he also believes plant-based vaccines still belong to the future.

“At this point in the road [plant-based vaccine researchers] have only expressed the protein in a few plants and only tested on small animals,” says Bhatnagar. “Plant vaccines are a long way off, because industry wants higher levels of productivity to be successful. Plus, everything requires approval from government regulators, so it all takes time. But, if I had to estimate, it might be ten years down the road.”

Daniell disagrees with this assessment, however. He says that vaccines against agents of bioterrorism are now on fast-track approval, and approval should come much sooner than 10 years.

A DOD spokesperson, who asked to remain anonymous, says that a plant-based anthrax vaccine would be of interest but that such a vaccine would have to be approved by the FDA. Also, says the spokesperson, “At present, the DOD has sufficient FDA-licensed anthrax vaccine to fulfill its policy. If the supply of anthrax vaccine was suddenly expanded, it might be that civilian purchasers of the vaccine would be less constrained than at present.”

## A Growth Industry?

Meanwhile, Daniell and his team aren’t content with producing 360 million doses of anthrax vaccine. Rather, they consider this a preliminary step towards an even greater goal: vaccines that are actually grown in and consumed along with a piece of fruit.

The idea of putting vaccines in plants or fruits was pioneered in 1992 by Charles Arntzen, currently codirector of the Center for Infectious Disease and Vaccinology at Arizona State University, after he observed a mother feeding her child a banana during a research trip to Thailand. Arntzen’s idea was simple: what if we could cut through the obstacles to vaccination by simply growing vaccines in fruit?

Many vaccines are hard to produce because of expensive fermenters, hard to ship because they often need to be kept refrigerated, and hard to distribute widely because it can take a trained health professional to administer the vaccine. All these factors make it particularly difficult to vaccinate populations in developing countries. Arntzen and his colleagues have continued exploring this line of thinking, and in the 1 March 2005 issue of *Proceedings of the National Academy of Sciences*, they conclude that a plant-based oral vaccine against hepatitis B, as delivered via potato, “should be considered as a viable component of a global immunization program.”

However, before we can eat bananas or potatoes for our booster shots, researchers need to figure out a few key problems.

First, there needs to be a way to standardize the vaccine’s dose. “Other vaccines are very exact on the dosages,” says Bhatnagar, “but with plant-based vaccines, what are you going to say? In the plant, levels might vary widely.”

The other major problem is that it takes months for a crop to grow, even a quick-growing one like tobacco, whereas the bacteria used in a fermentation system take only days or even hours. On the other hand, a crop system could be cheaper and produce more vaccine, compared to a fermentation system.

Despite the remaining hurdles, Daniell believes that his developments in tobacco plants will lead to an anthrax vaccine someday in the future. His team is also working on growing vaccine antigens against other diseases such as cholera, amebiasis, plague, and hepatitis C in tobacco plants.

“If a vaccine was produced in a plant cell, dried cells could be put in a capsule and delivered because the plant cell wall protects the protein already,” says Daniell. “Different delivery methods still need approval, but the cost of vaccines could drop from [up to] a hundred dollars to a few cents since basically all you are doing is powdering the plant and putting it in a capsule. For that reason, it is worth every regulatory hurdle, because it will pay off big time.”

## Figures and Tables

**Figure:**
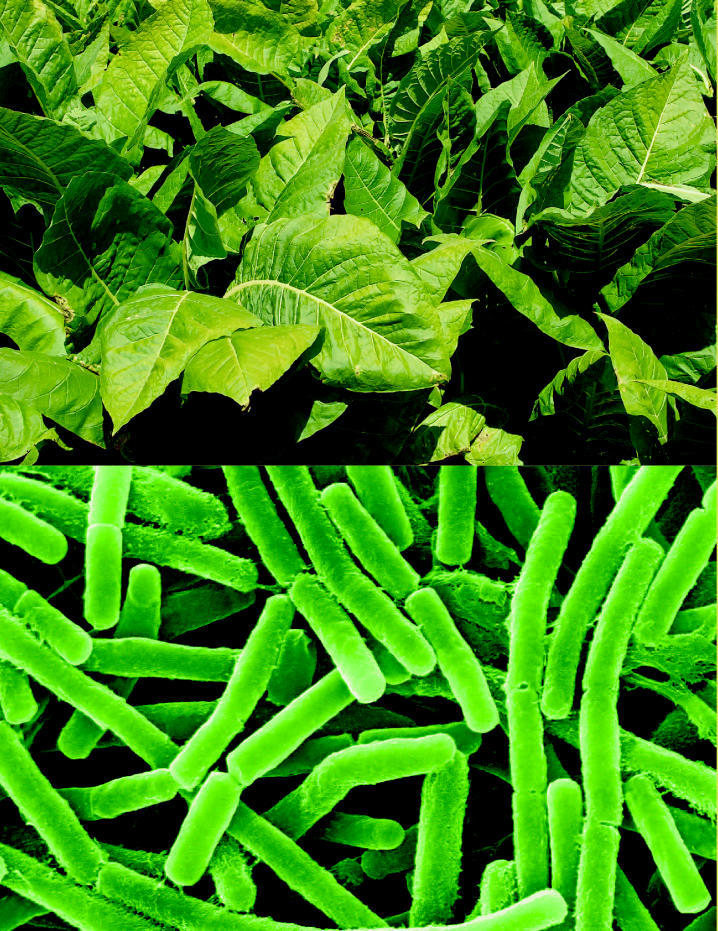


**Figure:**
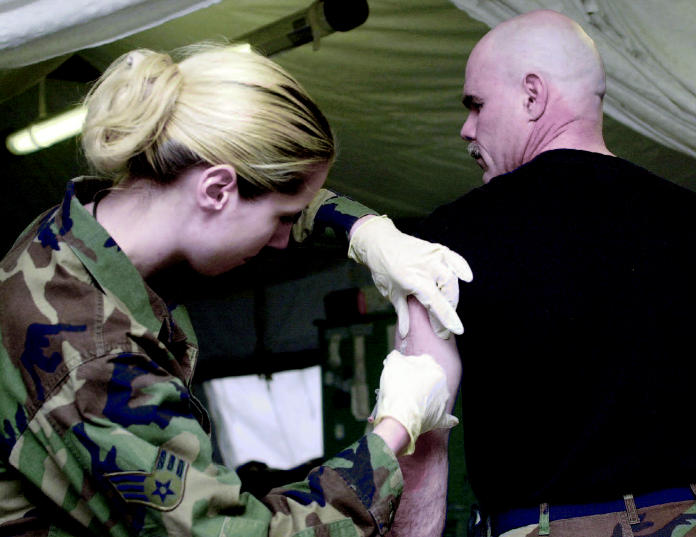
Fighters first, but then what? The government has stockpiled enough anthrax vaccine to supply military personnel but not nearly enough for public citizens. (above) Monica Carpenter, a medical services journeyman in the U.S. Air Force, administers anthrax vaccine to Technical Sergeant Ricky Anderson in Iraq.

**Figure:**
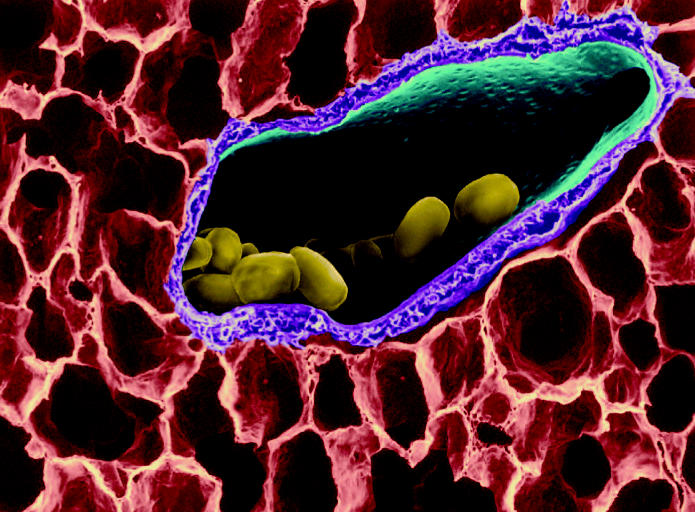
Sleeping giant? Although no one can predict if, when, or where an anthrax outbreak might occur, the magnitude of the threat makes the development of adequate vaccine resources a priority. (above) *Bacillus anthracis* spores in lung tissue.
