# Erratum

**Published:** 2006-07

A line of text was inadvertently omitted from the June 2006 Innovations article (“Plant vs. Pathogen: Enlisting Tobacco in the Fight Against Anthrax,” *EHP* 114:A364–A367 [2006]). The last sentence on page A365 should read: “The current anthrax vaccine works on this very principle by introducing nonvirulent PA into the body so antibodies are created.” *EHP* regrets the error.

